# *TaSPL17s* act redundantly with *TaSPL14s* to control spike development and their elite haplotypes may improve wheat grain yield

**DOI:** 10.3389/fpls.2023.1229827

**Published:** 2023-09-08

**Authors:** Hao Chen, Xing Zhang, Shuhao Xu, Chengxiang Song, Hailiang Mao

**Affiliations:** National Key Laboratory of Crop Genetic Improvement, Huazhong Agricultural University, Wuhan, China

**Keywords:** wheat, *TaSPL14s/17s*, spike development, haplotype, grain yield

## Abstract

Wheat is a staple crop for the world’s population, and there is constant pressure to improve grain yield, which is largely determined by plant architecture. SQUAMOSA promotor-binding protein-like (SPL) genes have been widely studied in rice, including their effects on plant architecture, grain development, and grain yield. However, the function of *SPL* homologous genes in wheat has not been well investigated. In this study, *TaSPL14s* and *TaSPL17s*, wheat’s closest orthologous of *OsSPL14*, were functionally investigated using gene-editing assays, revealing that these genes redundantly influence plant height, tiller number, spike length, and thousand-grain weight (TGW). Bract outgrowth was frequently observed in the hexa-mutant, occasionally in the quintuple mutant but never in the wild type. Transcriptome analysis revealed that the expression of many spike development-associated genes was altered in *taspl14taspl17* hexa-mutants compared to that in the wild type. In addition, we analyzed the sequence polymorphisms of *TaSPL14s* and *TaSPL17s* among wheat germplasm and found superior haplotypes of *TaSPL17-A* and *TaSPL17-D* with significantly higher TGW, which had been positively selected during wheat breeding. Accordingly, dCAPS and KASP markers were developed for *TaSPL17-A* and *TaSPL17-D*, respectively, providing a novel insight for molecular marker-assisted breeding in wheat. Overall, our results highlight the role of *TaSPLs* in regulating plant architecture and their potential application for wheat grain yield improvement through molecular breeding.

## Introduction

As wheat (*Triticum aestivum* L.) is a significant energy and protein resource for human beings, its production will have to increase by another 50% in the next 20 years to feed the growing world population (International Wheat Yield Partnership, https://iwyp.org/), making it imperative to improve wheat grain yield and quality. Plant architecture such as plant height, tiller number, tiller angle, and panicle morphology, is critical for grain yield in many cereal crops, including wheat. Due to the huge genome size and complicated genomic composition of hexaploid wheat, map-based cloning of yield-related genes is problematic. However, because of the close link between wheat and rice, researchers can clone homologous functional genes with a shared progenitor (Brenchley et al., 2012). To date, several genes regulating plant architecture and grain size have been identified and characterized in wheat, such as *TaGS3* (Yang et al., 2019), *TaGS5* (Ma et al., 2016), *TaGW2* (Liu et al., 2019), and *TaGW8* (Ma et al., 2019). The function and the molecular mechanisms of these genes in rice orthologs have been well documented and their effects on grain size appear to be shared by rice and wheat. These wheat genes have been linked to increased wheat productivity. More wheat genes that govern plant architecture and grain size would help us better understand the molecular mechanisms that regulate wheat productivity, which would be further used to enhance production.

*SQUAMOSA* promoter-binding protein-like (SPL) genes encode plant-specific transcription factors that play important roles in many developmental processes, such as phase change (Yamaguchi et al., 2009; Xu et al., 2016), plant architecture (Wang and Wang, 2017) and responses to biotic stress (Yin et al., 2019), root development (Yu et al., 2015). In cereal crops, most studies on *SPL* genes were involved in the regulation of plant architecture and grain yield. A maize domestication gene *TEOSINTE GLUME ARCHITECTURE* (*TGA1*), which belongs to the *SPL* family, was found to be responsible for the evolution of maize inflorescence architecture (Wang et al., 2005). In rice, *OsSPL7* was found to regulate the tiller number and modify plant architecture (Dai et al., 2018). *OsSPL13* positively regulated grain weight by regulating cell elongation, while *OsSPL16* increased the cell width to improve the grain weight (Wang et al., 2012; Si et al., 2016). *OsSPL14* (also known as *Ideal Plant Architecture 1*, *IPA1*) is considered to be a new “Green Revolution” gene that plays a critical role in constructing rice ideal plant architecture (Jiao et al., 2010; Miura et al., 2010; Wang and Wang, 2017). All these studies suggested the redundancy and specific function of *SPL* genes in plant architecture and grain size regulation. There are studies on the function of *TaSPLs* in wheat. *TaSPL13* regulates inflorescence architecture and development in transgenic rice and wheat (Li et al., 2020). The wheat orthologs of *OsSPL14* have been reported to target and activate *TaTB1* and *Barren Stalk1* (*TaBA1*) to regulate plant architecture and the strigolactone (SL) signaling pathway (Liu et al., 2017). Knockout *TaSPL14* reduced plant height, panicle length, spikelet number, and thousand-grain weight (TGW). However, the effects of these traits were relatively weak and the tiller number and panicle branching were not changed in *taspl14* triple mutant (Cao et al., 2021), while in *taspl7taspl15* hexa-mutant background, both the tiller number and spike length were affected (Pei et al., 2022). Whether other *SPL* genes function in plant architecture and spike development is not well known. Moreover, their functional redundancy in wheat needs to be investigated as only by understanding the fine regulation function of *SPL* genes can an ideal plant architecture of wheat be designed and wheat productivity be improved.

In this study, we isolated and characterized *TaSPL14* and its close homologs *TaSPL17* from wheat. Expression analyses showed that all six homolog genes of *TaSPL14/TaSPL17* were predominantly expressed in developing spikes. Hexa-mutants of *TaSPL14-aabbdd*_*TaSPL17*-*aabbdd* exhibited pleiotropic effects in plant height, tiller number and angle, panicle branching, and grain size. Furthermore, we conducted a haplotype analysis that showed two favorable haplotypes, *TaSPL17-A-A* and *TaSPL17-D-T*, which were significantly associated with grain width (GW) and TGW in wheat cultivars. Based on these haplotypes, we developed two molecular markers that could be useful for further wheat molecular breeding. In summary, our study provides insights into the functional roles of *TaSPL14* and *TaSPL17* homologs in wheat development and identifies promising haplotypes and molecular markers for improving grain traits in wheat breeding programs.

## Materials and methods

### Plant materials and phenotypic evaluation in wheat

The wheat variety Fielder was used for the transgene experiment in this study. The plants were cultured in a greenhouse with a 20 h/4 h light/dark photoperiod and a temperature regime of 22: 18°C (light: dark), or in an experimental field of Huazhong Agricultural University, Wuhan (30.47°N, 114.37°E). The heading date was calculated as days from the sowing date to the date when around half spikes were visible. The tiller number and angle were measured according to previous studies with minor modifications (Zhao et al., 2020). Briefly, more than eight individual plants for each genotype were randomly selected for phenotype analysis at the mature stage. The tiller angle was calculated by the arctangent function of ratios in a half-average d value to 30 cm, TA = 2 * arctan (d/2 * 30), in which d means the maximum distances (d in cm) among stems of each plant at 30 cm above the ground. Six spikes of the main stem from individual plants for each genotype were randomly selected to measure spike length (SL), spikelet number per spike (SNS), and grain number per spike (GNPS). Grain length (GL), grain width (GW), and thousand-grain weight (TGW) were evaluated using the intelligent test and analysis system (Wanshen Detection Technology Co., LTD., Hangzhou, China). For germplasm population, seed traits were obtained from plants grown in Xiangyang (32.17°N, 112.13°E) in the cropping season of 2019; Luoyang (4.82°N, 112.44°E) and Xiangyang in 2020; and Luoyang, Xiangyang, and Wuhan in 2021. The value of the best linear unbiased prediction (BLUP) for seed traits among different environments was calculated by the lme4 package in R 3.6.1 (http://www.r-project.org/). Each accession was planted in a 2-m single-row plot and a 25-cm distance between rows with a sowing rate of 35 seeds per row. The field experiments used a randomized complete block design with two replicates and the irrigation and other management of field trials were in accordance with local standard practices.

### Vector construction and wheat transformation

In total, six corresponding homolog genes of *TaSPL14s* (*TraesCS5A02G265900*, *TraesCS5B02G265600*, and *TraesCS5D02G273900*) and *TaSPL17s* (*TraesCS7A02G246500*, *TraesCS7B02G144900*, and *TraesCS7D02G245200*) were identified using the OsSPL14 amino acid sequence as a query in a BLAST search against the wheat IWGSC database (http://plants.ensembl.org/biomart). In order to simultaneously edit the genome sequence of *TaSPL14s* and *TaSPL17s*, two conserved single-guide RNAs (sgRNAs) were designed through the CRISPR direct online site (http://crispr.dbcls.jp/). The two sgRNAs were ligated into the intermediate vector pCBC-MT1T2 by PCR and subsequently inserted into the terminal vector pBUE411 as previously described (Xing et al., 2014). The construct was then transformed into Fielder by the *Agrobacterium*-mediated transformation method (Ishida et al., 2015). For transgenic plant and edited mutation detection, specific primers were designed to amplify the region covering the editing sites, and subsequent PCR sequencing was performed to select target-edited sites. The primers are listed in **Supplementary Table 1**.

### RNA sequencing and quantitative real-time PCR

For RNA sequencing, young spikes were collected at the double-ridge stage from the wild-type plants and the *taspl14taspl17* hexa-mutant line #2 with three biological replicates. The total RNA was extracted using Trizol according to the manufacturer’s protocol (Invitrogen). The quality and quantity of the total RNA were assessed using the Agilent 2100 Bioanalyzer System (Agilent Technologies, Santa Clara, CA, USA). The RNA library was constructed and subsequent sequencing was performed on the Novaseq 6000 sequencer (Illumina) with a pair-end sequencing strategy at Majorbio Technology Co., Ltd. (Shanghai, China). A total of 77.32 GB of clean data was obtained. The clean data of each sample was more than 11.55 GB, and the percentage of Q30 bases was more than 92.63%. The quality control of raw sequence data was performed using FastQC, and trimmed by Trimmomatic (Bolger et al., 2014). Afterward, high-quality cleaned reads were aligned to the wheat reference genome (IWGSC RefSeq v1.1, http://plants.ensembl.org/Triticum_aestivum/Info/Index) using Subread (Liao et al., 2013). The transcript read counts were normalized by transcripts per million reads (TPM). The R package “DESeq2” (Love et al., 2014) was used to perform differential expression gene analysis, and only the genes with |log2 fold change| > 1 and *p*-adjust < 0.01 were considered as differentially expressed genes (DEGs), which are listed in **Supplementary Table 3**.

For spatio-temporal expression pattern analysis of *TaSPL14s* and *TaSPL17s*, 10 different samples each with three replicates were collected, including root, stem, and leaves at the trefoil stage; stem base; flag leaves; tiller buds; young ears at the double ridge stage; young ears at glumes differentiation stage; and young spikes with 5-10 mm and 10-20 mm lengths. The total RNA was extracted and the reverse transcription was performed using a HiScript III 1st Strand cDNA Synthesis Kit (Vazyme R312-01) Quantitative real-time polymerase chain reaction (qRT-PCR) analyses were performed using the CFX96 real-time PCR detection system (Bio-Rad) in 10 μL reactions containing 1 μL cDNA template, each primer 0.25 μL, 5 μL Mix of SYBGR (TaKaRa), and 3.5 μL ddH_2_O. The relative expression level of each gene was obtained using the 2^-^ΔCT method (Livak and Schmittgen, 2001). The wheat *TaACTIN* gene (*TraesCS1A02G274400*) was used as an internal control. The primers used in qRT-PCR analysis are shown in **Supplementary Table 1**.

### Subcellular localization of the TaSPL17-D protein

To construct the subcellular localization vector for TaSPL17-D, the CDS of TaSPL17-D was amplified and subcloned into the pM999 vector driven by the CaMV 35S promoter (Song et al., 2020). The fusion construct was transformed into the *Agrobacterium* strain GV3101 and used for infiltration. Briefly, equal volumes of the fusion construct and H2B-mCherry, which was used as a nuclear localization marker (Mao et al., 2016), were mixed and coinfiltrated into the lower epidermis of tobacco leaves. The transfected plants were kept in a greenhouse for at least 48 h at 24°C. Fluorescence signals were visualized with LEICA SP8 laser confocal microscopy. The primers for vector construction are listed in **Supplementary Table 1**.

### Transcriptional activity assay of TaSPL17-D in yeast

Based on the predicted protein domains, TaSPL17-D was truncated into five fragments, including N-terminal, SBP domain, C-terminal, N-terminal with SBP, and SBP with C-terminal. They were separately in-frame fused to the binding domain (BD) of GAL4 in the pGBKT7 vector via homologous recombination. These recombinant constructs were transformed into yeast strain Y2H Gold with pGADT7 followed by selection on minimal synthetic dextrose medium SD/-Leu-Trp (SD medium lacking Leu and Trp) and SD/-Trp-Leu-His-Ade (SD medium lacking Trp, Leu, His, and Ade). The combinations of the pGADT7-T vector together with the pGBKT7-53 vector and the pGADT7-T vector together with the pGBKT7-lam vector were used as the positive control and the negative control, respectively. The initial OD value was adjusted to be 1, then 10 x, 100 x, and 1000 x serial dilutions were prepared to incubate at 28°C for 3 days and photographed. All culture media and reagents were purchased from Clontech (USA). Yeast transformation was performed according to the manufacturer’s protocol (Clontech, USA). The primers are listed in **Supplementary Table 1**.

### Electrophoretic mobility shift assay

The CDS of TaSPL17-D was cloned into the pGEX-6p-1 vector using homologous recombination reactions (Novagen, Madison, WI, USA). The construct was transformed into BL21 *Escherichia coli* cells (Weidi, Shanghai). The expression of the recombinant GST-TaSPL17-D protein was induced with 0.5 mM isopropyl beta-D-thiogalactopyranoside (IPTG) in 50 mL LB medium overnight at 16°C. Cells were harvested, washed, and suspended in 10 mL lysis buffer (Tris-HCl PH 8.0 50 mM, NaCl 400 mM, glycerol 10%), and then were sonicated for 0.5 h, containing 1 mM phenylmethylsulfonyl fluoride (PMSF) and 10 μL of β-mercaptoethanol. When the suspension became clear, the contents were centrifuged at 13,000 g for 20 min at 4°C, and the supernatant was collected and incubated with Glutathione Sepharose 4B beads (GE Healthcare, USA) for 2-3h at 4°C. The recombinant proteins were eluted five times with an Elution buffer. The purified proteins were stored at -80°C until further use.

The EMSA probes were commercially synthesized (Tsingke Biotechnology Co., Ltd). Primers were annealed to form a double strand by cooling from 95°C to room temperature (25°C) in an annealing buffer. The annealed products were subsequently constructed into the pGEM-T vector (Promega) The fluorescent probes were prepared with DY682 fluorescent modified vector universal primers (F: 5’-CATGGCCGCGGGATA-3’. R: 5’-GCGGCCGCACTAGTGAT-3’), and the non-fluorescent probes were prepared with unlabeled primers used as competitive probes with the same sequence as the labeled probes. Probes with core motif GTAC mutated into AAAA, were also prepared. The purified recombinant proteins and probes were then incubated in a tube with volume of 12 μL containing EDTA 1.13 mM, BSA 0.24 mg/mL, HEPES 7.22 mM, DTT 0.72 mM, Salmon sperm DNA 0.058 mg/mL, spermidine 1.27 mM, CHAPS 2.5%, glycerol 8%, probe 3.33 fmol/μL, protein 0.16-0.33 μL. Reactions were incubated on ice for 30-60 minutes and were then electrophoresed on 5% native polyacrylamide gel. The gel was photographed with the ChemiDoc Imaging System (BioRad). The probe sequences are listed in **Supplementary Table 1**.

### Transcriptional activity assays in *Nicotiana benthamiana*

The dual luciferase assay was performed as described previously (Guan et al., 2014). Briefly, the CDS of *TaSPL17-D* was cloned into pGreenII 62-SK to generate the effector vector. The 2.1-kb promoter sequence of *VRT-A2* was amplified from Fielder genomic DNA, and cloned into the reporter construct pGreenII 0800-LUC, containing the coding sequence of LUC from Renilla reniformis driven by the 35S promoter (Hellens et al., 2005), to generate the pGreenII reporter *pTaVRT-A2::LUC* plasmid. The following constructs were transformed into *Agrobacterium* strain GV3101: Empty vector (pGreenII 62-SK) and *pTaVRT-A2::LUC*, 35S::TaSPL17-D, and *pTaVRT-A2::LUC*. *Agrobacterium* strains containing these combinations of reporter and effector constructs were co-infiltrated into 4-week-old *N.benthamiana* leaves using an injection syringe. In order to compare the promoter activity of *TaSPL17-A* between *TaSPL17-A-A* and *TaSPL17-A-G* haplotypes, a 2 kb promoter sequence was amplified from Shanyou 225 for *TaSPL17-A-A* and Ribeiro for *TaSPL17-A-G*, and subcloned into pGreenII 0800-LUC. After 72h incubation, infected leaves were harvested and the relative LUC activity (LUC/REN) was quantified using the dual-luciferase reporter assay system kit on a GloMax-Multi luminescence reader (Promega). Normalized data were presented as the ratio of the value of luciferase activity to that of the control *35S::LUC* signal from four independent biological replicates. The primers for the constructs are listed in **Supplementary Table 1**.

### SNP confirmation and functional marker development

In order to confirm the SNP of *TaSPLs* among the germplasm, 20 wheat cultivars were randomly selected for sequencing (**Supplementary Table 6**). Gene-specific fragments were cloned into the TA/Blunt-Zero Cloning vector (Vazyme C601-01) and transformed into DH5α *E. coli* competent cells (WeiDi, Shanghai, China). Positive clones were sent for sequencing (Tsingke Biotechnology, Wuhan, China). Sequence alignments were performed via Snapgene software. The confirmed SNPs were used to develop either dCAPS or KASP markers. For the dCAPS marker for *TaSPL17-A*, gene-specific fragments were amplified by the primers and then separated by electrophoresis in 3% agarose gels after digestion by *Mn*II (New England Biolabs, Beverly, MA, USA). The KASP marker for *TaSPL17-D* was designed according to Wheatomics 1.0 (http://wheatomics.sdau.edu.cn/) (Ma et al., 2021). The reactions were in mixtures with volumes of 10 µL containing 1 µL of genomic DNA (50–100 ng), 5 µL of 2 × KASP master mix (V4.0, LGC Genomics), 2.5 µL ddH_2_O, and 0.5 µL of primers mix (10 µM of each allele-specific primer and 30 µM of common primer). PCR cycling was performed using the following procedure: hot start at 95°C for 10 min, followed by nine touchdown cycles (95°C for 15 s, touchdown at 61°C initially and decreasing by 0.6°C per cycle for 60 s), and followed by 38 additional cycles (95°C for 15 s, 55°C for 60 s). Three replicates for each genotype were performed. End-point fluorescence data were visualized and analyzed using CFX Maestro™ software (BIO-RAD, USA).

## Results

### Characterization of *TaSPL14s* and *TaSPL17s* in wheat

**Figure 1:**
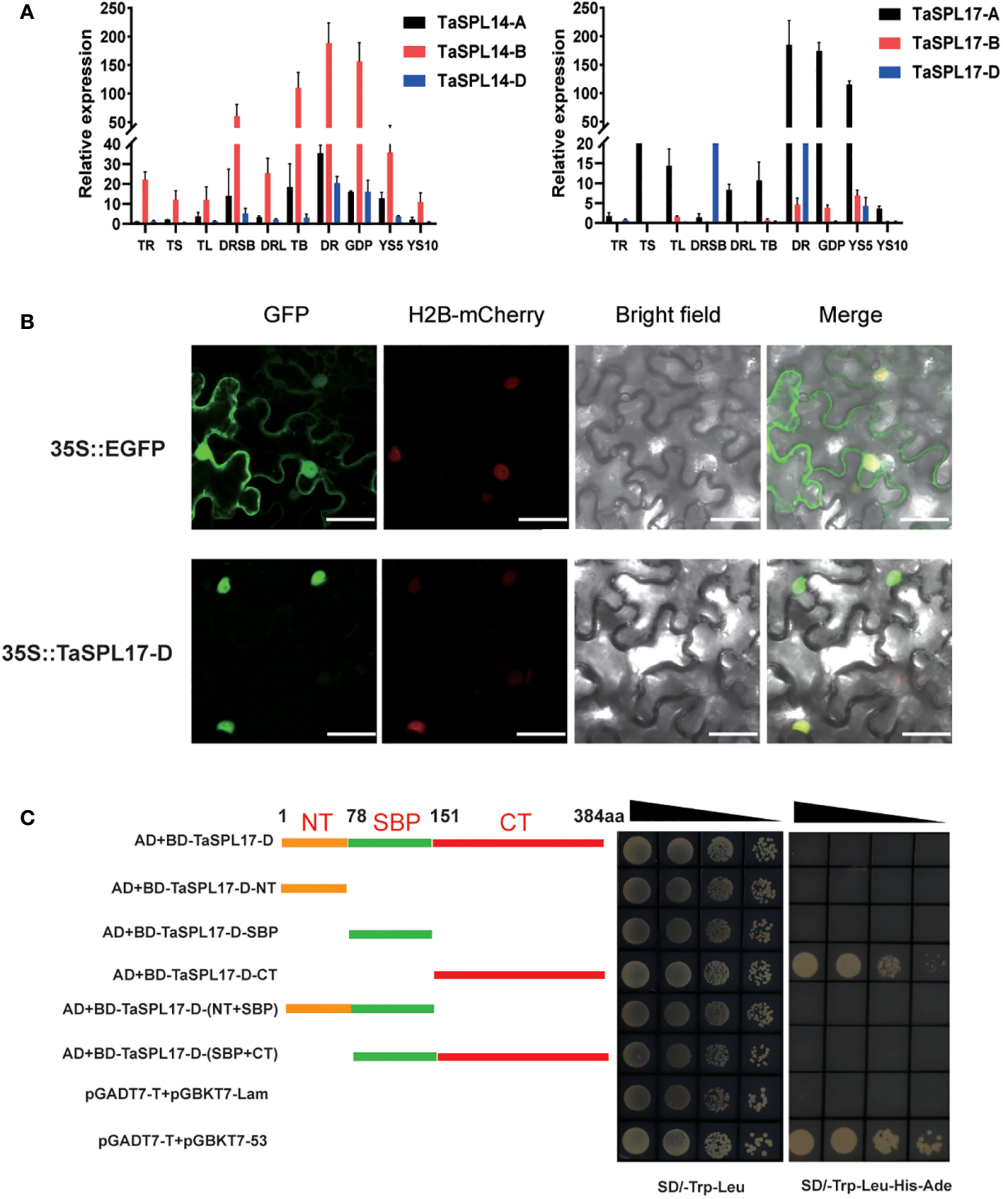
Characterization of *TaSPL14* and *TaSPL17* in wheat. **(A)** The expression pattern of six homoeologous *TaSPL14/17* genes in different tissues: TR, root at trefoil stage; TS, stem at trefoil stage; TL, leaves at trefoil stage root; DRSB, stem base at the double ridge stage; DRL, leaves at the double ridge stage; TB, tiller buds; DR, double ridge stage; GPD, glume primordium differentiation stage; YS5 and YS10 indicate young spike 5-10 mm and 10-20 mm in length, respectively. The relative expression levels were normalized to *TaACTIN*. Data were given as means ± SD of three biological replicates. **(B)** Subcellular localization of TaSPL17-D in tobacco leaf cells. Red fluorescence indicates the nuclear maker, H2B-mCherry. Scale bars = 50 μm. **(C)** The schematic representation of the coding sequences of constructs with one or more domain deletions. 1-77 aa, NT meaning N terminal; 78-150 aa, SBP domain; 151-384 aa, CT meaning C terminal; and aa indicates amino acids. The protein sequences of different lengths were fused to the BD vector. These recombinant plasmids were separately transformed into yeast strain Y2H Gold with pGADT7 followed by selection on minimal synthetic dextrose medium SD/-Leu-Trp (SD medium lacking Leu and Trp) and SD/-Trp-Leu-His-Ade (SD medium lacking Trp Leu His, Ade). The pGADT7-T vector together with the pGBKT7-53 vector and the pGADT7-T vector together with the pGBKT7-lam vector were used as the positive and negative control, respectively. The black triangles indicate serial dilution at 1 x, 10 x, 100 x, and 1000 x.

The phylogenetic tree of the SPL family genes in wheat has been constructed (Zhu et al., 2020; Cao et al., 2021; Li et al., 2022; Pei et al., 2022). Consistent with previous findings, we identified six genes, named *TaSPL14A/B/D* and *TaSPL17A/B/D* as the closest homologs of *OsSPL14* and *OsSPL17* in rice, and of *AtSPL9* and *AtSPL15* in *Arabidopsis*, which are crucial for panicle development in rice and floral induction in *Arabidopsis*, respectively (**Supplementary Figure 1A**, **Supplementary Table 2**) (Hyun et al., 2016). To investigate their role in spike development, we first examined the expression patterns of *TaSPL14-A/B/D* and *TaSPL17-A/B/D* in various stages of spike development (**Figure 1A**). All six genes displayed similar expression patterns with high expression levels in developing spikes, especially at the double ridge stage and glume differentiation stage. Their highly homologous protein sequences together with similar expression patterns suggested their possible functional redundancy (**Supplementary Figure 1B**). Furthermore, we also observed high expression in stem base and tiller buds, particularly for *TaSPL14-B*, indicating that *SPLs* may also affect vegetative growth. In addition, *TaSPL17-D* was randomly selected as a representative gene to determine the subcellular localization and transcriptional activity. The results showed that TaSPL17-D was, as expected, located in the nucleus, consistent with its role as a transcription factor (**Figure 1B**). We further tested its transcription activity in yeast and found that the C-terminal region of TaSPL17-D (TaSPL17-D-CT) contributed to its activity, however, the full length of TaSPL17-D showed no activity (**Figure 1C**). The N-terminal region, specifically the SBP domain, seemed to suppress the transcriptional activity of TaSPL17-D-CT (**Figure 1C**). These results together suggested that *TaSPL14s* and *TaSPL17s* have diverse expression profiles functioning as transcription factors in wheat.

### Identification of *taspl14s_taspl17s* wheat mutants

**Figure 2:**
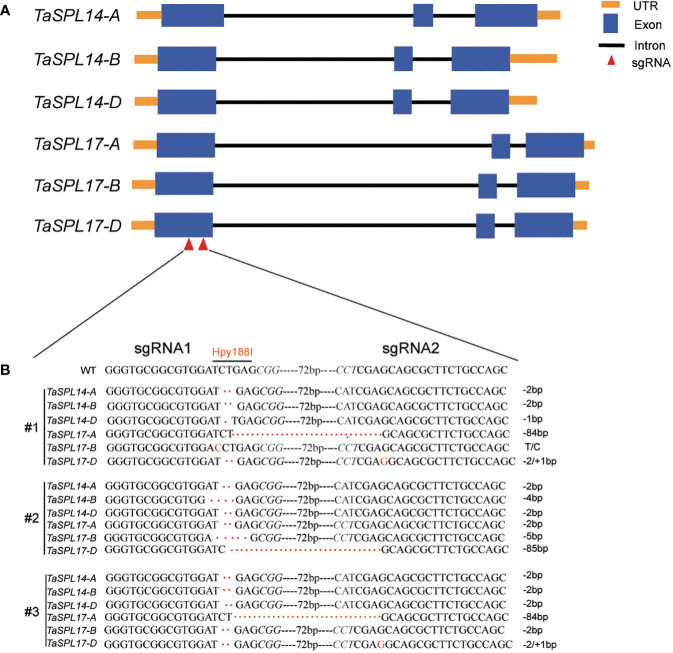
Gene editing of *TaSPL14/17* using CRISPR/Cas9. **(A)** Schematic diagram of six gene structures with orange boxes, UTR regions; blue boxes, exons; black solid lines, introns; red triangles, sgRNA positions. **(B)** The genotypes of three edited lines of *TaSPL14s* and *TaSPL17s* were identified by sequencing. Italic letters represent PAM (protospacer-adjacent motif) sites. The dashed box indicates the enzyme cut site; “--72bp--” indicates the sequence between two sgRNAs; “T/C” indicates base substitution in #1; “+/-” indicates base insertions or deletions, respectively.

To fully understand the function of *TaSPL14s* and *TaSPL17s* in spike and grain development, and their possible functional redundancy in wheat, we generated a *TaSPL14-aabbdd_TaSPL17-aabbdd* hexa-mutant using the CRISPR/Cas9 system (Xing et al., 2014). Two specific sgRNAs that target the conserved region of all six homologous genes of *TaSPL14* and *TaSPL17* were designed and constructed into a pBUE411 vector (**Figure 2A**). The construction was transformed into a wheat cultivar Fielder (Ishida et al., 2015), and 10 independent T_0_ lines were obtained. As a *TaSPL14s* triple mutant has been extensively investigated (Cao et al., 2021), we focused on the identification of hexa-mutant plants in which *TaSPL14s* and *TaSPL17s* were simultaneously edited without Cas9 confirmed by PCR. Further efforts to screen the T_2_ and T_3_ progenies led to the identification of two independent lines of *TaSPL14-aabbdd_TaSPL17-aabbdd* hexa-mutants. In these two lines, #2 and #3, all six genes were simultaneously mutated (**Figure 2B**), with a frameshift in the protein-coding sequences resulting in the inactivation of TaSPL14s and TaSPL17s proteins. In line #1, except for *TaSPL17-B* with one base substitution without amino-acid change, all the other five genes contained frameshift mutations caused by base insertion/deletion. Thus, line #1 was considered as a quintuple mutant in the following experiment to investigate the gene redundancy (**Figure 2B**).

### Agronomic traits characterization of *taspl14s_taspl17s* mutants

**Figure 3:**
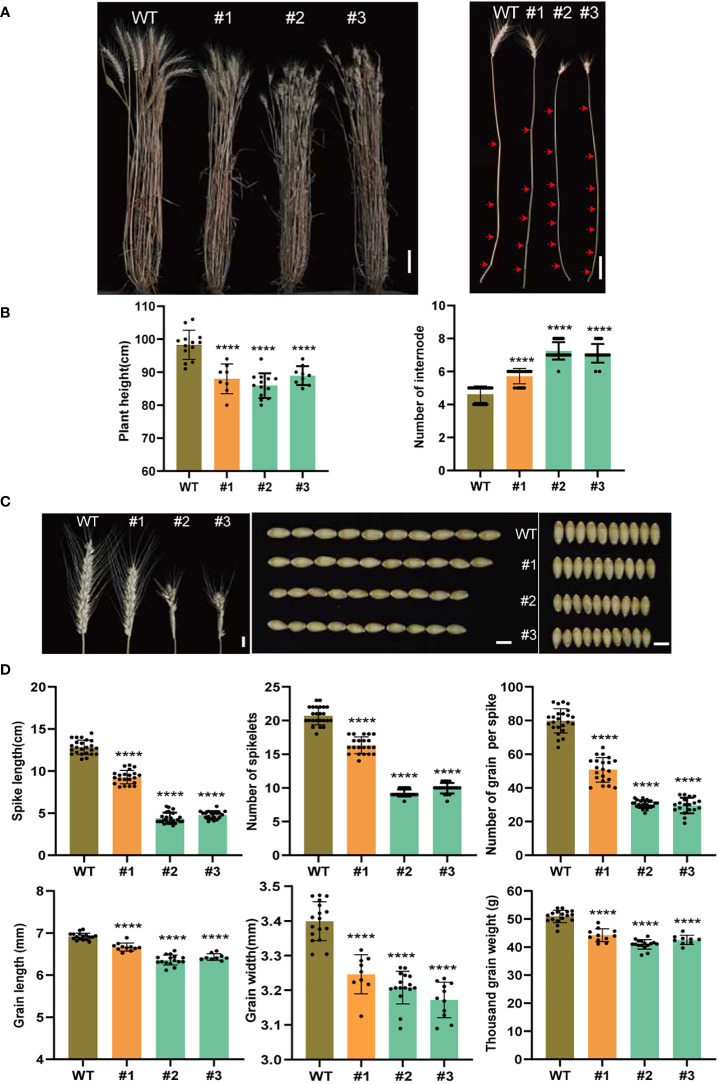
Phenotypic analysis between different genotypes of mutant and wild-type plants. **(A)** Representative images to show plant height (left) and stem internode (right). #1 indicates quintuple mutant material; #2 and #3 represent hexa-mutant plants; WT represents Fielder; red arrows indicate nodes. Scale bar = 10 cm, **(B)** Representative images to show a spike, grain length, and grain width of wild type and edited lines, Scale bar = 1 cm. **(C, D)** Statistical analysis for plant height and the number of elongated internodes **(C)** for spike length, number of spikelets per spike, grain number per spike, grain length, grain width, and thousand-grain weight **(D)** of wild type and mutants, n > 10. All data were given as means ± SD, and significant difference was determined by Student’s *t*-test. ****, *p* < 0.0001.

Three lines, one quintuple- (#1, *TaSPL14-aabbdd_TaSPL17-aaBBdd*) and two hexa- (#2, #3, *TaSPL14-aabbdd_TaSPL17-aabbdd*) mutants, and the wild-type plants were grown in the field to characterize the agronomic traits in detail. Compared to the wild-type plants, both the quintuple- and hexa-mutant plants showed a significant decrease in plant height (**Figure 3A**). However, the number of elongated internodes increased in all three mutant lines (**Figure 3B**). This indicated the dwarf phenotype of the mutants was due to shortened internodes. Higher tiller numbers but a smaller tiller angle was also observed in both the quintuple- and hexa-mutants (**Supplementary Figures 2A-C**). We checked several known tiller number and angle-related genes and found all of these genes were downregulated in the mutants, coinciding with their negative regulations of either tiller number or angle (**Supplementary Figure 2D**). As *TaSPL14s* and *TaSPL17s* are mainly expressed in young spikes, we investigated the phenotypic effects of spikes after knocking out both *TaSPL14s* and *TaSPL17s.* Compared to the wild-type plants, both the quintuple and hexa-mutants of *TaSPL14s* and *TaSPL17s* showed a significant decrease in spike length, number of spikelets per spike, and number of grains per spike (**Figure 3C**). Compared to that of hexa-mutants, the effects of *TaSPL14-aabbdd_TaSPL17-aaBBdd* quintuple-mutants were generally much weaker, suggesting their apparent redundancy roles in spike development (**Figures 3C, D**). Moreover, we frequently observed the outgrowth of bract on the base of spikes in the *TaSPL14-aabbdd_TaSPL17-aabbdd* hexa-mutants, but this was rarely seen in the *TaSPL14-aabbdd_TaSPL17-aaBBdd* quintuple mutants and was absent in the wild-type plants (**Supplementary Figure 3**). To investigate whether *TaSPL14s* and *TaSPL17s* regulate wheat grain development, grain width (GW), grain length (GL), and thousand-grain weight (TGW) were evaluated in all three mutant lines. The results showed that all three mutant lines had a significant decrease in GW, GL, and TGW (**Figures 3C, D**). Taken together, these results suggest that *TaSPL14s* and *TaSPL17s* have pleiotropic positive roles in the regulation of plant height, spike architecture, and grain productivity while having a negative role in tiller number and tiller angle.

### *TaSPL14/TaSPL17* regulated spike development by targeting MADS-box genes

**Figure 4:**
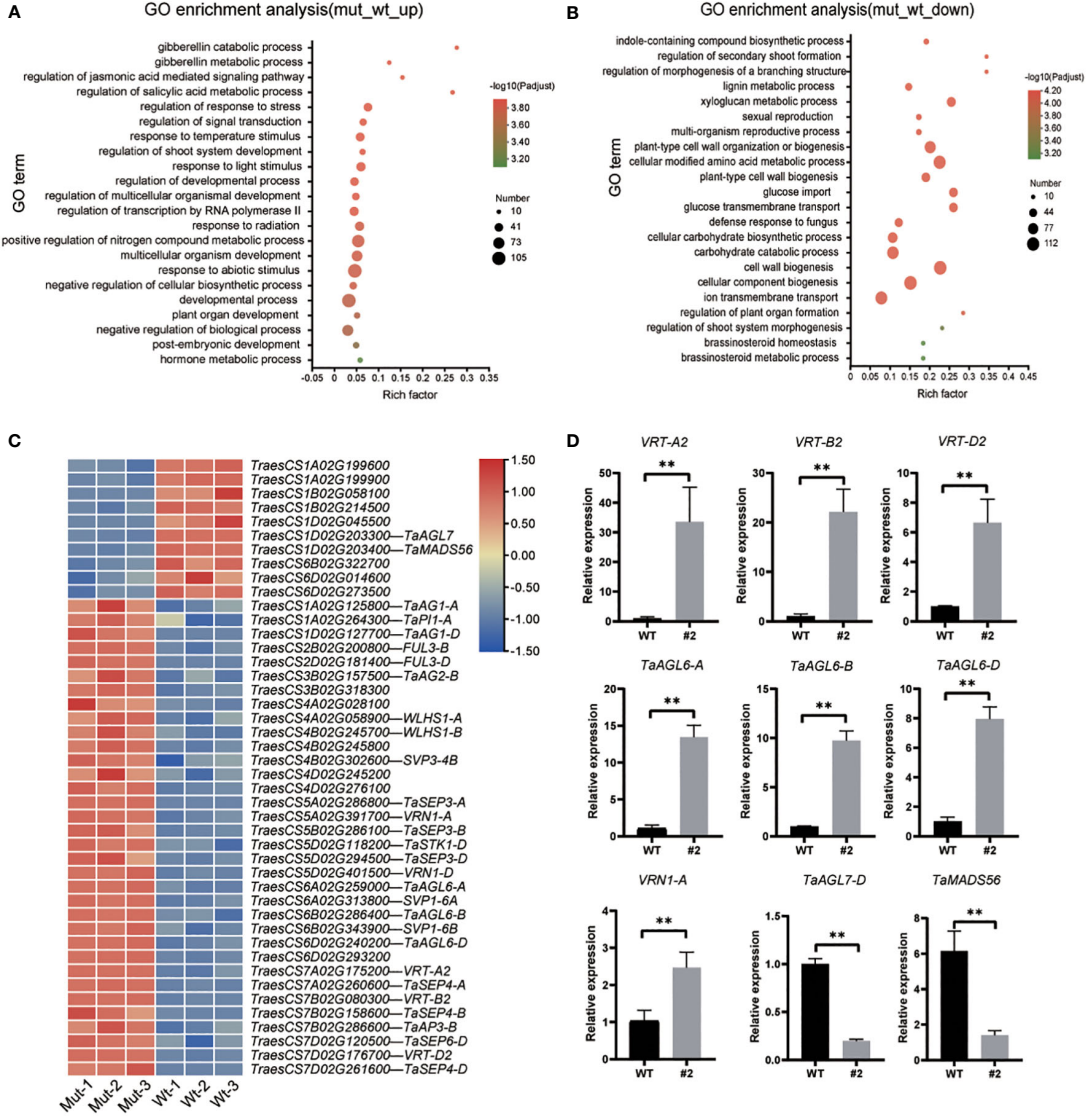
GO enrichment analysis of wild-type and mutant differentially expressed genes (DEGs) and validation by qRT-PCR. GO enrichment analysis of mutant up-regulated **(A)** and down-regulated **(B)** DEGs. The colors indicate -log10 (*P* adjust values) of the GO enrichment. The size of the circle indicates the number of enriched genes. **(C)** Heatmap of expression levels of MADS-related genes in differentially expressed genes. **(D)** qRT-PCR validation of differentially expressed genes. The expression level was normalized to that of *TaACTIN*. Data were given as means ± SD of three biological replicates. Significant difference was determined by Student’s *t*-test. **, *P* < 0.01.

RNA-seq experiments were performed to explore the regulatory network of *TaSPL14/TaSPL17* in spike development. According to their expression patterns, the young panicle at the double-ridge stage was collected from *taspl14taspl17* hexa-mutant line #2 and wild-type plant Fielder (WT; **Supplementary Figures 4A, B**). The correlation between the three biological replicates was greater than 0.93, indicating that the transcriptome data were reproducible and could be used for subsequent analysis (**Supplementary Figure 4C**). A total of 7,683 differentially expressed genes (DEGs; |log2 fold change| > 1, *p* adjust < 0.01) were identified in *taspl14taspl17* compared to WT, with 2128 genes upregulated and 5555 genes downregulated (**Supplementary Figure 4D**). Gene ontology (GO) enrichment analysis showed that the upregulated genes were mainly associated with the gibberellin catabolic process, regulation of shoot system development, and developmental progress, while the down-regulated genes were mainly linked to the indole-containing compound biosynthetic process, cell wall biogenesis (**Figures 4A, B**; **Supplementary Table 4**). The GO items of different genes partially overlapped previous studies on the *taspl14* triple mutant (Cao et al., 2021), further supporting the function redundancy of *TaSPL14s* and *TaSPL17s.*

**Figure 5:**
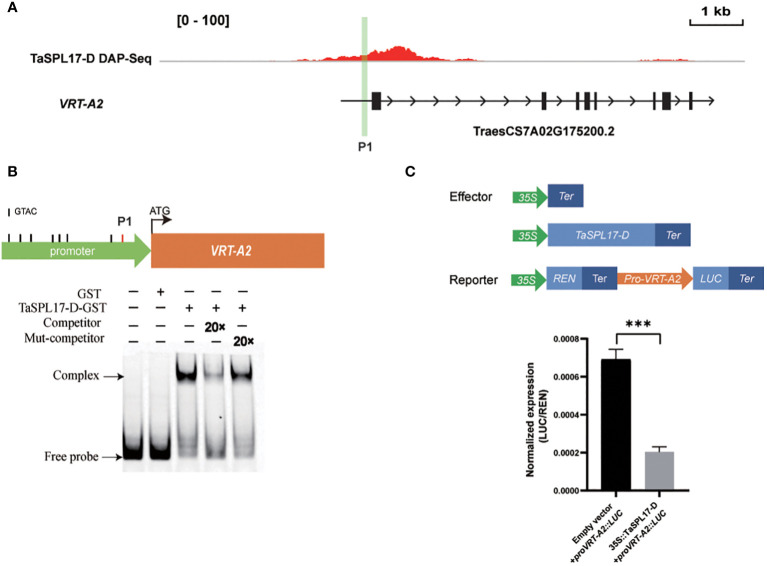
TaSPL17-D directly binds to the promoter of *VRT-A2* and inhibits its expression. **(A)** TaSPL17-D binding the promoter of *VRT-A2* was identified by DAP-seq data. The black box indicates the gene exon. The black arrow indicates the direction of the gene. P1 represents the binding site for EMSA. Bar = 1 kb. **(B)** Electrophoretic mobility shift assay (EMSA) shows that TaSPL17-D binds to promoter sequences (*VRT-A2-P1*) containing the GTAC motifs *in vitro*. The black solid line represents the GTAC motif, and the red solid line represents the EMSA-verified GTAC binding site. Fluorescent probes are added to all lanes, and “-/+” represents the presence and absence of corresponding proteins and probes. “20 x” indicates 20-fold molar excesses of the competitor or mutated competitor (Mut-competitor) probes relative to the concentration of the fluorescent probe. The mut-competitor probe represents a change of the binding motif GTAC to AAAA. **(C)** Transactivation assays with *N. benthamiana* leaves showed that TaSPL17-D repressed the transcription of *VRT-A2*. Relative LUC activity (luciferase (LUC)/Renilla luciferase (REN)) was measured at 60 h after infiltration. Each bar in the graph corresponds to the mean value ± SD of four independent replicates. Significant difference was determined by Student’s *t*-test. *** *p* < 0.001.

As the striking phenotype of spike development in the hexa-mutant, we checked the transcription of genes from the MADS-box family, which is well-known to be involved in the regulation of flower development and flowering time (Klein et al., 1996). We found 44 MADS-box genes were significantly changed in *taspl14taspl17* with 34 upregulated and 10 downregulated (**Figure 4C**; **Supplementary Table 5**), several of which were further verified by quantitative RT-PCR (qRT-PCR) analysis (**Figure 4D**). Some of these MADS family-like genes, such as *VRN1*, *FUL3*, and *SVP1*, have previously been shown to be involved in flower development and heading date (Li et al., 2019). Intriguingly, we actually observed around 2 days for quintuple and 6 days for hexa-mutant plants delay of heading when compared to that of WT in the field, respectively (**Supplementary Figure 5**). Considering the defects of panicle and tiller in the *taspl14taspl17* mutants (**Supplementary Figure 4**), we determined whether TaSPLs directly regulated *TaMADS55*/*VRT-A2* which has been reported to play dual roles in both panicle and tiller development (Adamski et al., 2021; Liu et al., 2021; Mao et al., 2022). We analyzed the promoter sequence of *VRT-A2* and identified a GTAC core binding motif of SPL (Cao et al., 2021; Pei et al., 2022) (**Figure 5A**). Therefore, an electrophoretic mobility shift assay (EMSA) was performed and the result showed that TaSPL17-D could directly bind the *VRT-A2* promoter (**Figure 5B**). Moreover, TaSPL17-D also could repress the expression of reporter gene *firefly luciferase* (*LUC*) driven by the 2.1 kb promoter of *VRT-A2* in *N. benthamiana* leaves (**Figure 5C**). These results suggested that TaSPL17-D directly bound and repressed *VRT-A2* expression and that SPL may regulate spike development through *VRT-A2* in wheat. Sequence rearrangement in the intron-1 region of *VRT-A2* resulted in elevated gene expression and the long glume phenotype (Adamski et al., 2021; Liu et al., 2021). Although striking enhanced expression of *VRT-A2* in hexa-mutant, we did not observe long glume, suggesting other factors might be needed to produce long glume while these unknown factors were somehow suppressed in hexa-mutant.

### *TaSPL14/TaSPL17* superior haplotypes are positively associated with grain yield in wheat

**Figure 6:**
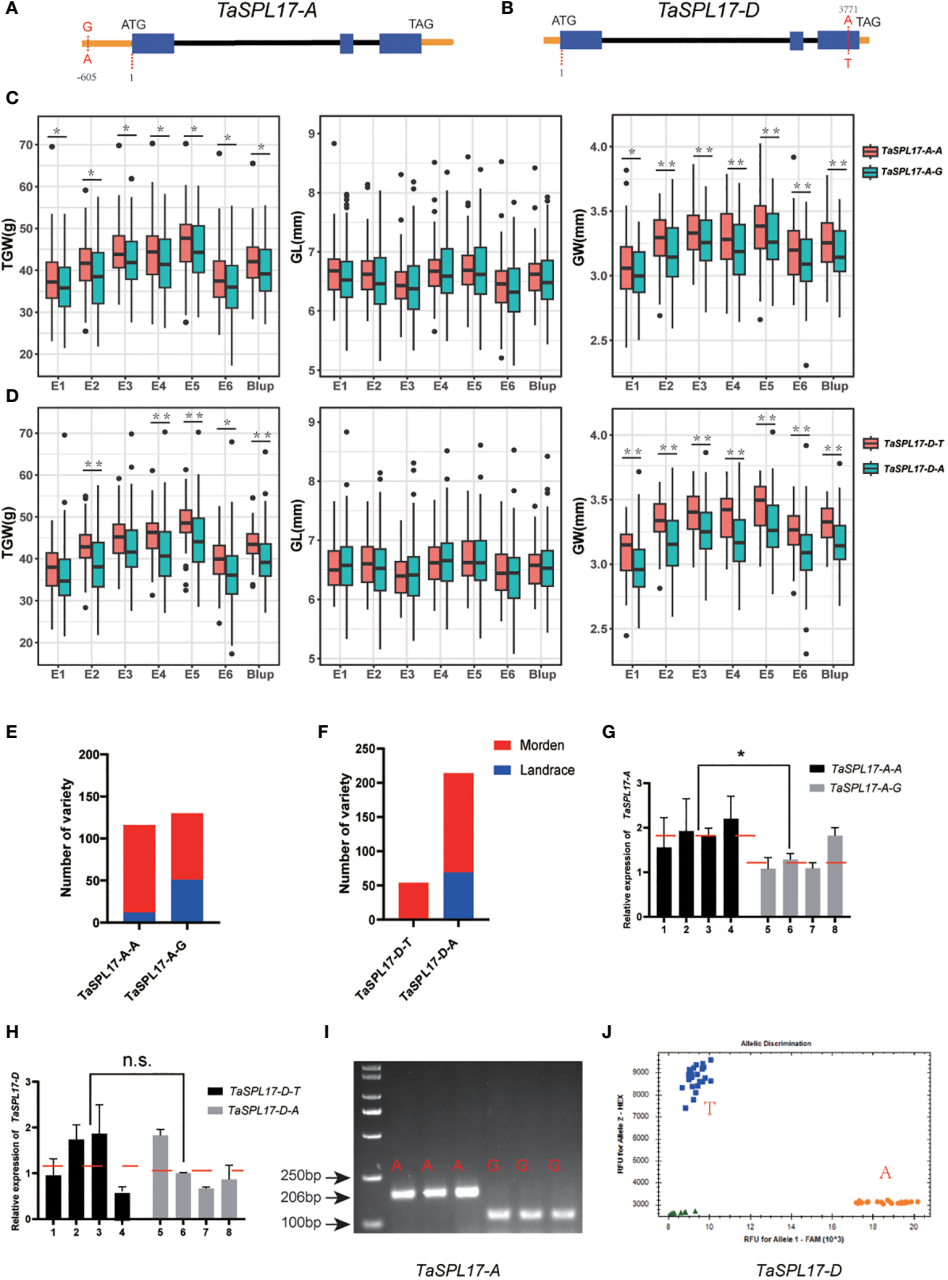
Haplotype analysis and functional marker development of *TaSPL17-A*, *TaSPL17-D*. Gene structures of *TaSPL17-A* **(A)** and *TaSPL17-D* **(B)** and two SNPs were identified based on re-sequencing of germplasm, both of which showed significant association with grain width and thousand-grain weight. Comparative phenotypic analysis of thousand-grain weight, grain length, and grain width of two haplotypes of *TaSPL17-A* **(C)** and *TaSPL17-D* **(D)** in six environments. E1-E3, seeds harvested from Xiangyang in 2019, 2020, and 2021 crop seasons, respectively; E4-E5, seeds from Luoyang in 2020 and 2021, respectively; E6, seeds from Wuhan in 2021. Blup indicates the best linear unbiased estimate values among six environments. Significant difference was determined by Student’s *t*-test. *, *p* < 0.05; **, *p* < 0.01. Frequencies of two haplotypes of *TaSPL17-A* **(E)** and *TaSPL17-D* **(F)** in 326 germplasm. **(G, H)** indicates *TaSPL-7A/D* expression level in different haplotypes, respectively. The red dotted lines show the average expression levels of each haplotype accessions. 1-4 indicates JZ034, JZ125, JZ252, and JZ304, respectively. 5-8 indicates JZ135, JZ267, JZ288, and JZ362, respectively. Data were given as means ± SD of three biological replicates. Significant difference was determined by Student’s *t*-test. *, *P* < 0.05. n.s. indicates no significant difference. **(I)** A dCAPS marker was developed based on *TaSPL17-A* SNP. The PCR products were digested with restriction enzyme *Mn*II and separated on 3% agarose gel. A represents the variant genotype and G indicates the reference genotype the same as Chinese spring. **(J)** Validation of the KASP maker for *TaSPL17-D*. T represents the variant genotype and A indicates the reference genotype. Green triangles are the water used as a negative control.

To explore the correlation between natural variations in *TaSPL14/TaSPL17* and agronomic traits, the polymorphisms were analyzed in the coding and 2-kb promoter regions of *TaSPL14/TaSPL17* in 326 wheat germplasm genotyped by re-sequencing (CNP0003712, https://db.cngb.org/). 3 and 1 SNPs were found in the promoter of *TaSPL14-A* and *TaSPL14-B*, respectively, as well as 11 (2 in exon, 9 in promoter), 9 (1 in exon, 8 in promoter), 1 (in exon) SNPs were found in *TaSPL17-A*, *TaSPL17-B*, *TaSPL17-D*, respectively. We then performed an association analysis between these polymorphisms and three agronomic traits, grain width (GW), grain length (GL), and thousand-grain weight (TGW), collected from six environments during the cropping seasons from 2020–2022 together with the best linear unbiased estimate (BLUP) values. The results showed none of the polymorphisms in *TaSPL14s* were significantly associated with either GW or TGW. However, we found two single nucleotide polymorphisms (SNPs) were significantly associated with GW and TGW traits in all six environmental growth conditions (*P* < 0.05). One was located in the promoter of *TaSPL17-A* (-605bp, A/G) and the other one was located in the coding region of *TaSPL17-D* (3771bp, A/T) which resulted in an amino acid change from Asn to Tyr (**Figures 6A, B**). The results showed *TaSPL17-A-A* and *TaSPL17-D-T* haplotypes were favorable alleles when compared to *TaSPL17-A-G* and *TaSPL17-D-A*, respectively, as evidenced by a significant increase in GW and TGW (**Figures 6C, D**). None of the polymorphisms were found to be associated with grain length (**Figures 6C, D**). Furthermore, the wheat varieties with two superior haplotypes had the highest thousand-grain weight and grain width, and the effect of the *TaSPL17-A-A* superior haplotype was over that of *TaSPL17-D-T* as no grain weight or grain width difference was observed between AT (*TaSPL17-A-A/TaSPL17-D-T*) and AA (*TaSPL17-A-A/TaSPL17-D-A*) (**Supplementary Figure 6B**).To determine the selection characteristics of the *TaSPL17* haplotypes in wheat breeding, we assessed the variations of *TaSPL17-A* and *TaSPL17-D* haplotypes in 326 wheat accessions (**Supplementary Table 6**) and found that the frequency of *TaSPL17-A-A* and *TaSPL17-D-T* haplotypes was much higher in modern cultivars than that in landraces (**Figures 6E, F**). These results suggest that both *TaSPL17-A-A* and *TaSPL17-D-T* underwent positive selection in wheat breeding processes. As *TaSPL17-A-A* was in the promoter region, we assumed that it might confer a higher grain weight by elevating gene transcription. To this end, we compared the expression level of *TaSPL17-A* in grain collected at seven days post-anthesis from eight wheat varieties with different haplotypes. It was shown that the *TaSPL17-A* expression level was significantly higher in the *TaSPL17-A-A* accessions than in the *TaSPL17-A-G* accessions (**Figure 6G**). We then compared the promoter activity of two haplotypes using transient expression experiments in *N. benthamiana* leaves. Consistently, the promoter activity of *TaSPL17-A-A* was significantly higher than that of *TaSPL17-A-G* (**Supplementary Figure 6A**). No expression difference was detected between accessions with *TaSPL17-D-T* and *TaSPL17-D-A* haplotypes, suggesting the haplotype effect of *TaSPL17-D* might not result from transcriptional level differentiation but the amino acid change of the encoding protein (**Figure 6H**). The polymorphisms of these two SNPs were further confirmed by PCR sequencing. Although KASP markers are preferred for their high throughput and easy automated analysis, we were unable to design a KASP marker for *TaSPL17-A* after several attempts. Instead, we developed a dCAPS marker to distinguish between *TaSPL17-A-A* and *TaSPL17-A-G* (**Figure 6I**). Nevertheless, we successfully designed a KASP marker to accurately differentiate the haplotypes between *TaSPL17-D-A* and *TaSPL17-D-T* (**Figure 6J**). These two markers can be used to pyramid favorable alleles in developing high-yield varieties in the future.

## Discussion

The plant-specific transcription factor SPLs were first reported to regulate flower development in *Antirrhinum majus* (Klein et al., 1996). Since then, efforts have been made to main gene function in different plant species, especially in rice, where these transcription factors have diverse functions in plant growth and development, and grain yield which is easy to attract attention for main crop plants (Wang and Zhang, 2017). The breakthrough came with the identification of *OsSPL14*, also known as *IPA1/WFP* (Jiao et al., 2010; Miura et al., 2010). The *ipa1* mutant, with a point mutation to perturb the cleavage of *OsSPL14* mRNA by *miR156*, shows an ideal plant architecture with increased plant height and panicle branches, and decreased tiller number (Jiao et al., 2010). Later on, *GW8/OsSPL16* was identified to regulate rice grain size, shape, and quality (Wang et al., 2012). *GLW7/OsSPL13* was found to positively regulate cell size, resulting in increased grain size and yield (Si et al., 2016). Although 56 *SPL* genes were identified in allohexaploid wheat (Zhu et al., 2020), only a few of them have been functionally analyzed in wheat. While wheat and rice *SPL* homologs have shown similar functions, functional differentiation may have occurred (Li et al., 2022). For example, knockout plants of *TaSPL14* showed reduced plant height, spike length, and TGW, which is similar to that of *OsSPL14* function in rice. However, the tiller number of *TaSPL14* knockout plants, in contrast to *OsSPL14*, was unaffected, which could be the gene function differentiation in different organisms (Cao et al., 2021). Alternatively, this result could be due to gene redundancy, considering the complex genome of hexaploidy wheat. Therefore, in this study, we established both quintuple- and hexa-mutants of *TaSPL14s* and *TaSPL17s*, both of which showed the closest homolog to *OsSPL14*. We found that the hexa-mutant showed a striking phenotype in reducing plant height, spike length, and TGW to a greater extent than that of the *TaSPL14s* triple mutant (Cao et al., 2021). The phenotype of the quintuple mutant was much weaker than that of the hexa-mutant, further indicating function redundancy. The tiller number of both the quintuple and hexa-mutants was significantly increased compared to WT, which was not found in the *TaSPL14* triple mutant. This might suggest that *TaSPL14s* redundantly regulate the tiller number together with *TaSPL17s*. Furthermore, the tiller angle was also changed in the mutant plants and this was further evidenced by checking the relevant gene expression. To our knowledge, this has not been reported for *SPL* function although we do not know exactly how this happens at present. More work needs to be done to gain full comprehension of this tiller angle regulation, which is also a key trait for ideal plant architecture construction. Intriguingly, we frequently found bract outgrowth in the hexa-plant which is usually repressed, as also reported in rice (Wang et al., 2021), showing functional conservation among different organisms.

*TaSPL14-aabbdd_TaSPL17-aabbdd* hexa-mutants exhibited a striking phenotype characterized by abnormal spike development, suggesting that *TaSPL14s/TaSPL17s* are involved in both heading date and spike development. Consistently, transcriptomic analysis comparing the mutants to the wild type revealed significant changes in 44 MADS-box genes (**Figure 4C**; **Supplementary Table 5**). Among them, 34 genes were up-regulated including *VRN1*, *FUL3*, and *VRT2*, which have been well characterized to be involved in both heading date and spike development (Li et al., 2019; Li et al., 2021). This suggests that *TaSPL14s/TaSPL17s* may act as a key regulator upstream of these MADS-box transcription factors. It is known that *VRN1* and *FUL2* are essential to promote the transition from IM to TS and for spikelet development (Li et al., 2019). The defective spike development in *TaSPL14-aabbdd_TaSPL17-aabbdd* hexa-mutants, characterized by a short spike with much fewer spikelets compared to wild type, may be due to the elevated expression of *VRN1* and *FUL3* in hexa-mutants. However, the delayed heading date in the mutants, despite the upregulation of *VRN1*, suggests that other repressed components may be necessary for flowering. Further investigation is needed to unravel this puzzle. Another upregulated gene in the mutants, *VRT2*, has been reported to play pleiotropic roles in both panicle and tiller development in wheat (Adamski et al., 2021; Liu et al., 2021) and rice (Mao et al., 2022). Despite observing higher expression of *VRT2* in hexa-mutants, we did not observe the elongated glumes or grains reported in previous studies (Adamski et al., 2021; Liu et al., 2021). It’s possible that other unidentified components were required for glume elongation, but they were suppressed in the mutants. Nonetheless, we confirmed that TaSPL17 could directly bind to the promoter of *VRT2* and repress its expression. Furthermore, we found that *AGL6* genes, known to repress flowering, were up-regulated in the *TaSPL14-aabbdd_TaSPL17-aabbdd* hexa-mutants (Kong et al., 2022). This may explain the delayed heading date in the mutants. However, in terms of spikelet development, the upregulation of AGL6 seems inconsistent, as it was shown to promote the spikelet number (Kong et al., 2022). These results suggest *TaSPL14/17* may coordinately regulate heading date and spikelet development, but they also have independent effects. We also observed a significant increase in the transcript levels of floral organ identity genes which have been documented in the ABCDE model of flower development in *Arabidopsis* (Theissen et al., 2016), such as A-class *VRN1*, B-class *TaAG1*, and E-class genes *TaSEP3*, *TaSEP4* and *TaSEP6* (**Figure 4C**). Overall, these findings suggest that *TaSPL14s/TaSPL17s* act as master genes for spike development. However, due to its pleiotropic and complex effects on plant architecture, inferring their function in spike development solely based on transcriptome data is insufficient, and further investigation into the genetic pathway of spike development is needed.

Various alleles of *OsSPL14* have been discovered and utilized in rice breeding (Jiao et al., 2010; Zhang et al., 2017). Single allele *OsSPL14^ipa1^* introduction has resulted in a 10% increase in grain yield in field conditions (Jiao et al., 2010). Another allele *OsSPL14/WFP*, which is involved in epigenetic regulation, has also been introduced into different rice varieties to enhance the grain yield (Ashikari and Matsuoka, 2006; Miura et al., 2010; Kim et al., 2018). In contrast, few favorable alleles of *TaSPLs* have been reported, despite the possibility that some superior alleles may have been selected during wheat breeding. This may be due to the lack of available markers. Despite the significant increase in wheat yield through artificial selection, the narrow genetic basis of wheat germplasm in current breeding programs has hindered substantial improvement in yield in the past decade (Fu and Somers, 2009; Mir et al., 2012; Ray et al., 2013). Developing molecular markers for essential genes could assist greatly in fueling wheat yield increment, and this also becomes urgent considering the growing population. In our assay, we examined the polymorphisms of *TaSPL14/17*. We identified 4 SNPs in *TaSPL14s* and 21 in *TaSPL17s* identified in promoters and exons. Based on our resequencing data, we could not find polymorphism in the coding region of *TaSPL14s*. However, we did find two superior haplotypes of *TaSPL17-A-A* and *TaSPL17-D-T* and found that the two haplotypes had been positively selected during the wheat breeding processes. Consequently, we developed two molecular markers for future application in high-yield wheat breeding. Our results not only enhance our understanding of SPL function in wheat but also offer valuable resources for wheat molecular breeding.

## Data availability statement

The datasets presented in this study can be found in online repositories. The names of the repository/repositories and accession number(s) can be found below: https://www.ncbi.nlm.nih.gov/, PRJNA973251.

## Author contributions

HC performed most of experiments, analyzed the data, prepared the figures and tables, and wrote the original draft. XZ, CS, and SX performed some of the experiments and material planting and data analysis. HM was in charge of conceptualization, supervision, writing, and funding acquisition. All authors contributed to the article and approved the submitted version.

## Supplementary material

Supplementary Figure 1 Phylogenetical tree construction and sequence analysis. **(A)** Phylogenetical tree construction using SPL protein sequences from rice, wheat, and Arabidopsis. The genome of rice, wheat, and Arabidopsis contains 19, 56, and 16 SPL genes, respectively, clustered into 11 subfamilies (G1 to G11). The phylogenetic tree was constructed using MEGA-X with the neighbor-joining method, and the bootstrap test was performed with 1000 replicates. **(B)** Amino acid sequence alignment of the homologous genes of the G4 subgroup, the black box sequence represents the SBP domain, and the sequence comparison analysis was performed by DNAMAN.

Supplementary Figure 2 Phenotypic analysis of tiller number and tiller angle and verification of related gene expression by qRT-PCR. **(A)** Representative images to show the tiller number and tiller angle difference between WT (Fielder) and edited plants. The letter d indicates the maximum distance among stems of single plants at 30 cm (h) above ground level. Statistical analysis of tiller number **(B)** and tiller angle **(C)** phenotype. More than 8 individual plants for each genotype were randomly selected for phenotype analysis. Data were given as means ± SD. Significant difference was determined by Student’s *t*-test. **, *p* < 0.01. ****, *p* < 0.0001. **(D)** qRT-PCR verification of tiller number and tiller angle related gene expression in tiller buds. Data were presented as means ± SD, *n* = 3. Student’s *t*-test was used to determine significant difference. *, *p* < 0.05. **, *p* < 0.01.

Supplementary Figure 3 Representative images to show bract leaves in mutant plants. Red rows indicate bract leaves in lines #2 and #3, which is not found in WT (Fielder) but occasionally in line #1. Bar = 1 cm.

Supplementary Figure 4 RNA-Seq analysis of young spikelet at the double-ridge stage. **(A)** Young spike at the double-ridge stage, Bar = 1 mm. **(B)** Plants at the double-ridge stage with WT left and #2 right. Bar = 10 cm. **(C)** Correlation analysis of transcriptome data. **(D)** Differentially expressed genes between hexa-mutant (#2) and wild-type were shown in a volcano plot.

Supplementary Figure 5 Phenotypic analysis at the heading stage. **(A)** Representative images of Wild-type and mutant plants grown in the field. Bar = 10 cm. **(B)** Statistical analysis of heading date. Significant difference was determined by Student’s *t*-test. *, *p* < 0.05. ****, *p* < 0.0001.

Supplementary Figure 6 Promoter activity of *TaSPL17-A* and the genetic interaction between *TaSPL17-A* and *TaSPL17-D*. **(A)** The promoter of each haplotype was cloned into the pGreenII 0800-LUC vector, and the promoter activity was measured based on the LUC/REN ratio. The values were given as means ± SD. *n* = 5. **, *p* < 0.01. **(B)** AT, AA, GT, GA indicate wheat accessions with *TaSPL17-A-A* and *TaSPL17-D-T*, *TaSPL17-A-A* and *TaSPL17-D-A*, *TaSPL17-A-G* and *TaSPL17-D-T, TaSPL17-A-G* and *TaSPL17-D-A*, respectively. Different letters mean significant difference (*p* < 0.05) determined by Student’s *t*-test.
