# Supplementary figures and images for: TaSPL17s act redundantly with TaSPL14s to control spike development and their elite haplotypes may improve wheat grain yield
Source: Front Plant Sci. 2023 Sep 8;14:1229827. doi: 10.3389/fpls.2023.1229827 (PMC10514913; doi:10.3389/fpls.2023.1229827)

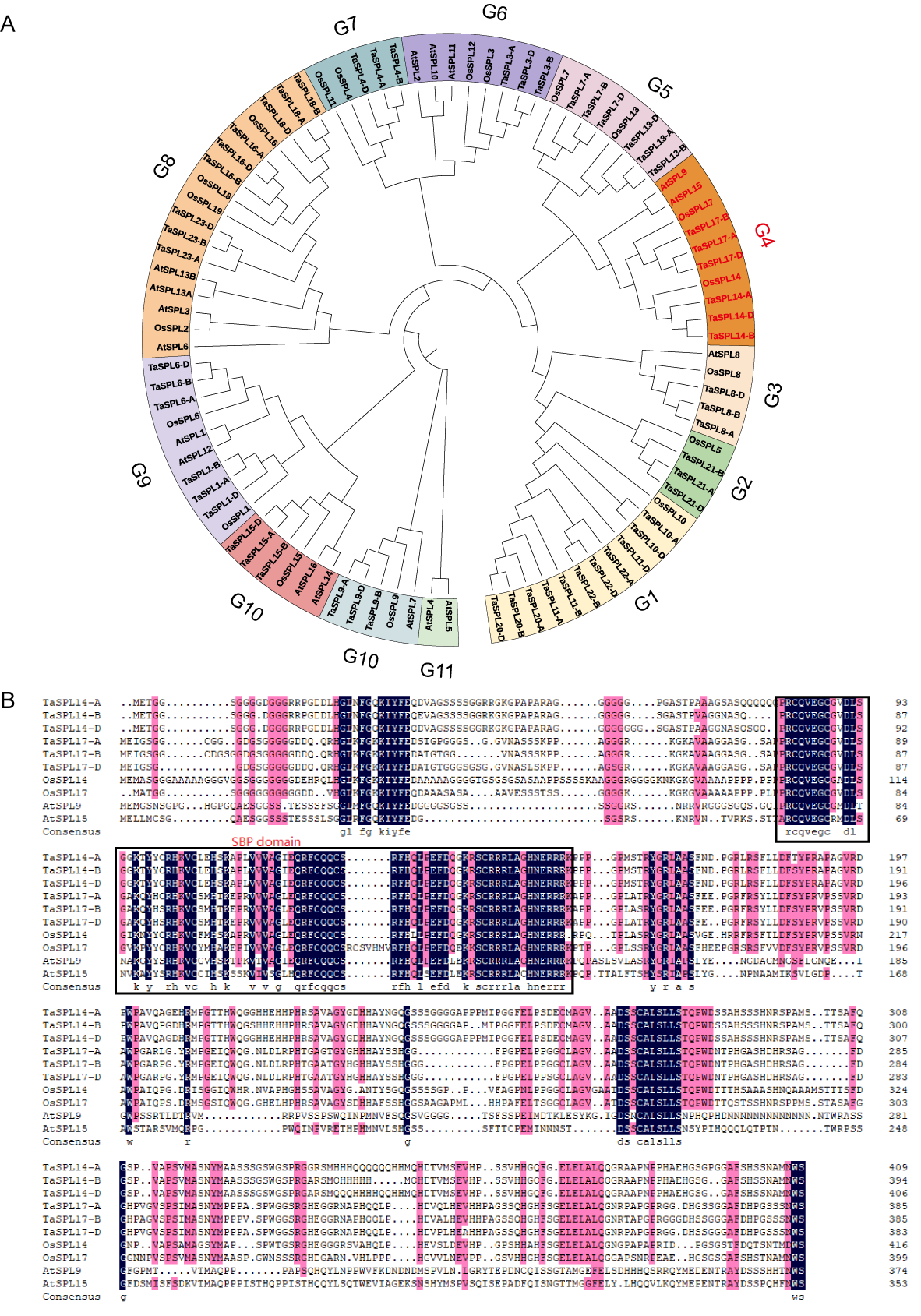

Supplement: Supplementary Figure 1 — Phylogenetical tree construction and sequence analysis. (A) Phylogenetical tree construction using SPL protein sequences from rice, wheat, and Arabidopsis. The genome of rice, wheat, and Arabidopsis contains 19, 56, and 16 SPL genes, respectively, clustered into 11 subfamilies (G1 to G11). The phylogenetic tree was constructed using MEGA-X with the neighbor-joining method, and the bootstrap test was performed with 1000 replicates. (B) Amino acid sequence alignment of the homologous genes of the G4 subgroup, the black box sequence represents the SBP domain, and the sequence comparison analysis was performed by DNAMAN. [file Image_1.tif]

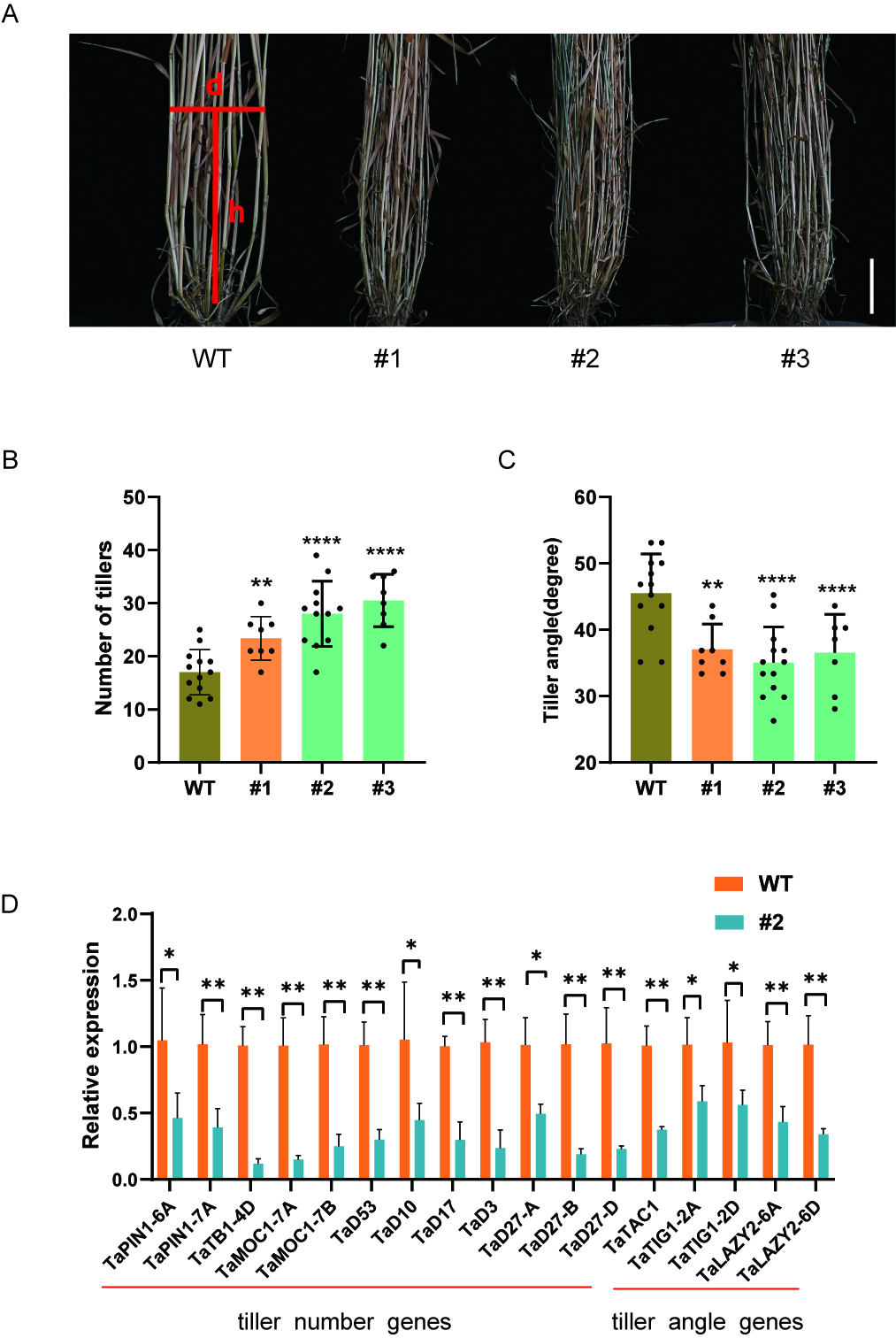

Supplement: Supplementary Figure 2 — Phenotypic analysis of tiller number and tiller angle and verification of related gene expression by qRT-PCR. (A) Representative images to show the tiller number and tiller angle difference between WT (Fielder) and edited plants. The letter d indicates the maximum distance among stems of single plants at 30 cm (h) above ground level. Statistical analysis of tiller number (B) and tiller angle (C) phenotype. More than 8 individual plants for each genotype were randomly selected for phenotype analysis. Data were given as means ± SD. Significant difference was determined by Student’s t-test. **, p < 0.01. ****, p < 0.0001. (D) qRT-PCR verification of tiller number and tiller angle related gene expression in tiller buds. Data were presented as means ± SD, n = 3. Student’s t-test was used to determine significant difference. *, p < 0.05. **, p < 0.01. [file Image_2.tif]

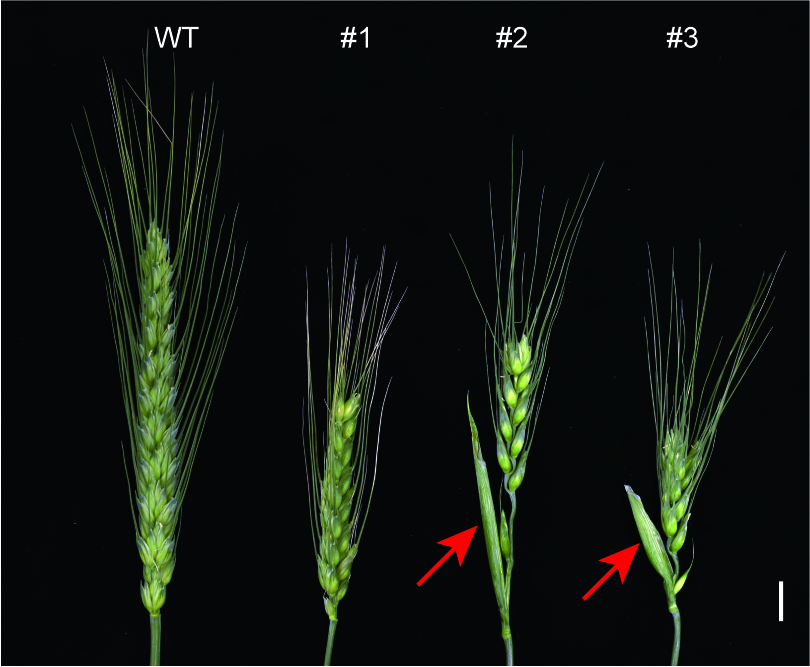

Supplement: Supplementary Figure 3 — Representative images to show bract leaves in mutant plants. Red rows indicate bract leaves in lines #2 and #3, which is not found in WT (Fielder) but occasionally in line #1. Bar = 1 cm. [file Image_3.tif]

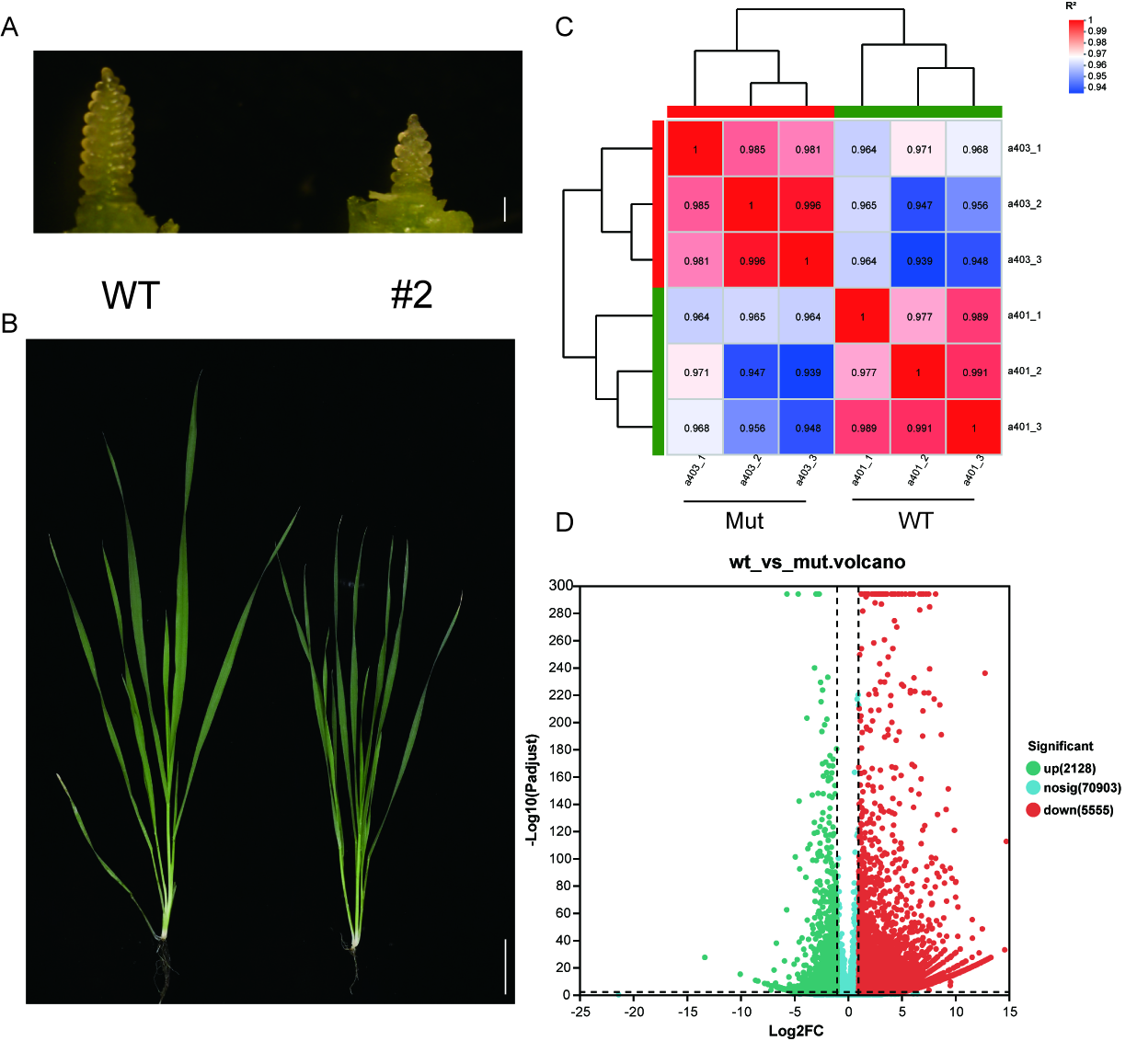

Supplement: Supplementary Figure 4 — RNA-Seq analysis of young spikelet at the double-ridge stage. (A) Young spike at the double-ridge stage, Bar = 1 mm. (B) Plants at the double-ridge stage with WT left and #2 right. Bar = 10 cm. (C) Correlation analysis of transcriptome data. (D) Differentially expressed genes between hexa-mutant (#2) and wild-type were shown in a volcano plot. [file Image_4.tif]

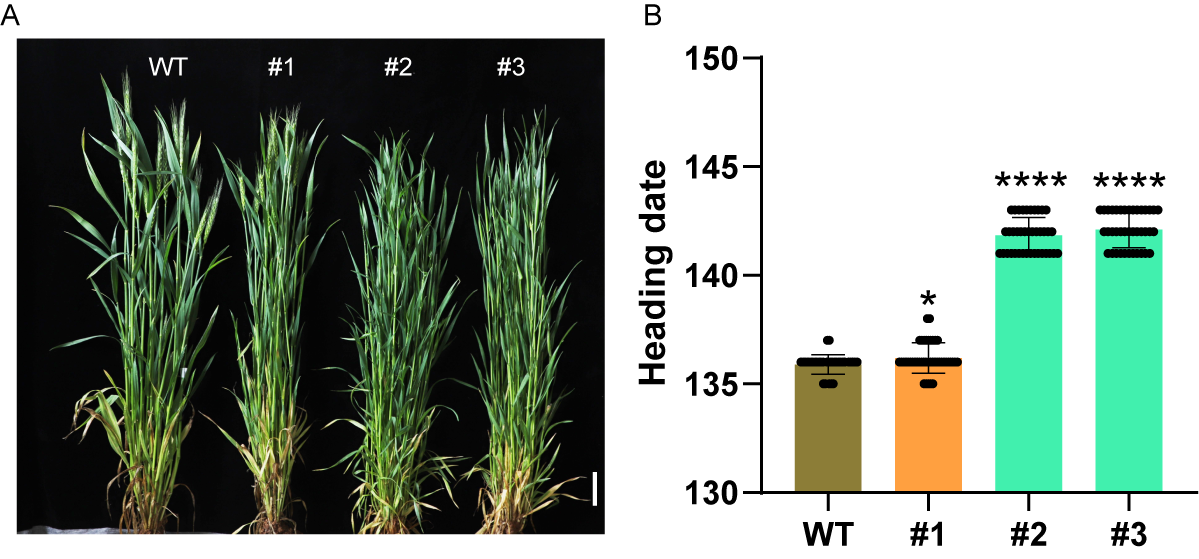

Supplement: Supplementary Figure 5 — Phenotypic analysis at the heading stage. (A) Representative images of Wild-type and mutant plants grown in the field. Bar = 10 cm. (B) Statistical analysis of heading date. Significant difference was determined by Student’s t-test. *, p < 0.05. ****, p < 0.0001. [file Image_5.tif]

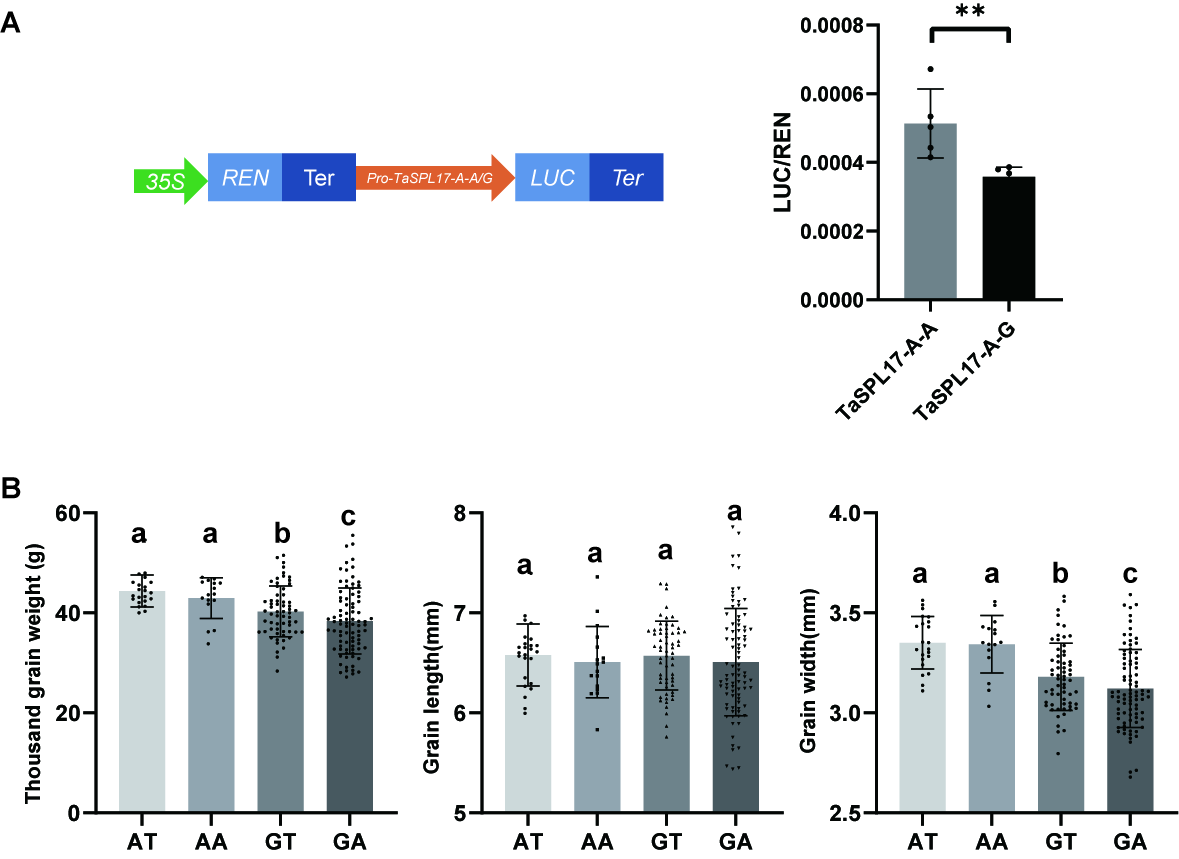

Supplement: Supplementary Figure 6 — Promoter activity of TaSPL17-A and the genetic interaction between TaSPL17-A and TaSPL17-D. (A) The promoter of each haplotype was cloned into the pGreenII 0800-LUC vector, and the promoter activity was measured based on the LUC/REN ratio. The values were given as means ± SD. n = 5. **, p < 0.01. (B) AT, AA, GT, GA indicate wheat accessions with TaSPL17-A-A and TaSPL17-D-T, TaSPL17-A-A and TaSPL17-D-A, TaSPL17-A-G and TaSPL17-D-T, TaSPL17-A-G and TaSPL17-D-A, respectively. Different letters mean significant difference (p < 0.05) determined by Student’s t-test. [file Image_6.tif]
